# Properties and identification of antibiotic drug targets

**DOI:** 10.1186/1471-2105-11-195

**Published:** 2010-04-20

**Authors:** Tala M Bakheet, Andrew J Doig

**Affiliations:** 1 Faculty of Life Sciences, The University of Manchester, Michael Smith Building, Oxford Road, Manchester M13 9PT, UK; 2 Manchester Interdisciplinary Biocentre, The University of Manchester, 131 Princess Street, Manchester M1 7DN, UK

## Abstract

**Background:**

We analysed 48 non-redundant antibiotic target proteins from all bacteria, 22 antibiotic target proteins from *E. coli *only and 4243 non-drug targets from *E. coli *to identify differences in their properties and to predict new potential drug targets.

**Results:**

When compared to non-targets, bacterial antibiotic targets tend to be long, have high β-sheet and low α-helix contents, are polar, are found in the cytoplasm rather than in membranes, and are usually enzymes, with ligases particularly favoured. Sequence features were used to build a support vector machine model for *E. coli *proteins, allowing the assignment of any sequence to the drug target or non-target classes, with an accuracy in the training set of 94%. We identified 319 proteins (7%) in the non-target set that have target-like properties, many of which have unknown function. 63 of these proteins have significant and undesirable similarity to a human protein, leaving 256 target like proteins that are not present in humans.

**Conclusions:**

We suggest that antibiotic discovery programs would be more likely to succeed if new targets are chosen from this set of target like proteins or their homologues. In particular, 64 are essential genes where the cell is not able to recover from a random insertion disruption.

## Background

Infectious and parasitic diseases caused by pathogenic microorganisms, including bacteria, viruses and fungi, are major threats to human health. In particular, diseases commonly result from exposure to gram positive bacteria, such as *Staphylococcus aureus*, *Streptococcus pneumoniae *and *group A Streptococcus*, and gram negative bacteria, such as *E. coli *and *Helicobacter pylori*. Antibacterial drugs are the major weapons to kill bacteria or suppress their activity. Due to the inevitable evolution of antibiotic resistance, the development of novel antibiotics is essential.

Antibiotics work either by stopping bacterial growth or by killing the bacteria, without harming the human host. The following are the most common modes-of-action of antibiotics:

(1) Inhibit synthesis of peptidoglycan. These antibiotics work by interfering with the synthesis of bacterial cell walls by either: blocking the transport of peptidoglycan monomers synthesized in the cytosol across the cytoplasmic membrane, inhibiting a transpeptidase and hence the formation of the peptide cross-links, or blocking both the transglycosidase and transpeptidase enzymes. The transglycosidases are essential for the formation of glycosidic bonds between sugars and transpeptidases are essential for the formation of peptide cross-links [1].

(2) Alter the microbial cytoplasmic membrane. The polymixins are cationic peptides consisting of a cyclic peptide with a fatty acid chain. The interaction between the cationic peptide and the membrane causes disruption of the bacterial cell membrane and increases the permeability of cell components [2].

(3) Alter translation. Many antibiotics work by binding to bacterial ribosomes. Examples of antibiotics that work by binding to the 30S ribosomal subunit are aminoglycosides and tetracyclines, which prevent the binding of tRNA [3,4]. Other macrolide antibiotics, such as erythromycin, bind to the 50S ribosomal subunit and block the exit tunnel of the bacterial ribosome [5].

(4) Inhibit nucleic acid replication by blocking topoisomerases that are essential for supercoiling, bacterial DNA replication and separation of circular bacterial DNA. The fluoroquinolone antibiotic class contains potent inhibitors for topoisomerases or DNA gyrase [6].

(5) Inhibit transcription. Some antibiotics, such as rifampin or rifampicin, work by binding to RNA polymerase and inhibiting the transcription of DNA to mRNA [7].

After the first widespread use of antibacterial drugs in the 1940s, bacterial pathogens started to develop resistance to existing drugs, particularly after excessive antibiotic use. The three basic mechanisms of bacterial resistance to antibiotics are: (1) Production of an enzyme to inactivate the antibiotic, such as a β-lactamase to hydrolyse penicillin. (2) Mutation in the target site receptor of the enzyme or the ribosomal subunit that leads to ineffective drug binding. (3) Alteration in transport proteins to prevent antibiotic entry or promote active efflux from the cell [8]. There is thus an urgent need to discover new strategies to discover and develop effective antibiotic drugs to overcome widespread and growing antibiotic resistance. One way in which this may be achieved is by identifying bacterial proteins that may be the targets for new classes of antibiotics. Sakharkar *et al. *addressed this problem by identifying essential bacterial genes (i.e. essential for the growth, replication, viability, or survival of the microorganism) that have no human homologues[9].

Here, we determine key properties of antibiotic target proteins and use machine learning to identify new potential targets.

## Results

### Primary Sequence, Secondary Structure and Post-translational Properties

All the proteins in the targets and non-targets data sets were analysed for their primary sequence properties and post-translational modifications. As all features showed a non-normal distribution using the Kolmogorov-Smirnov test, p-values were calculated using the Mann-Whitney test. Table 1 shows the differences between the mean values of length, hydrophobicity, secondary structure, transmembrane helices (TMHMM), SignalP, low complexity regions (LCR), pI, amino acid preferences and post-translational properties for targets and non-targets. Targets tend to be larger proteins with mean lengths of 420 and 515 amino acids for bacteria and *E. coli *respectively compared to 316 for non-targets. Bacterial targets show significant preferences for Pro and Val and disfavour Trp, Met, Leu and Gln. Secondary structure analysis shows that bacterial targets have less α-helix and more β-Sheet. The percentage of target helices is 32% compared to 42% for non-targets with significant p-values (all bacterial p-value = 0.00027; *E. coli *p-value = 0.015). The prediction of transmembrane helices shows that targets tend to have fewer helices (0.3 for all bacterial and 0.5 for *E. coli*) compared to 1.4 for non-targets. Antibiotic and *E. coli *targets are less hydrophobic (-93 for *E. coli *and -85 for all bacterial) compared to non-targets (-21) with significant p-values (all bacterial p-value = 0.049 *E. coli *p-value = 0.067). No statistically significant differences were observed for pI, SignalP, NetNGlyc and NetOGlyc and LCR between the bacterial or *E. coli *targets and non-targets. This indicates that these features do not contribute to the druggability of a bacterial protein. This is expected for glycosylation, as the glycosylation site prediction programs are optimised for use in metazoa; indeed the predicted frequencies for this property are low. Nevertheless, it is possible to use any sequence feature (including these) for machine learning.

**Table 1:** Mean amino sequence features and p-values for all bacterial targets, *E. coli *targets and non-targets.

Feature	Non-targets	Bacterial targets	p-value comparing all targets to non-targets	*E. coli *targets	p-value comparing *E. coli *targets to non-targets
Length	316	420	0.032	515	0.001
% Trp	1.52	0.98	0.005	0.74	6 × 10^-5^
% Met	2.95	2.54	0.099	2.26	0.015
% Val	7.11	7.85	0.0002	7.94	0.017
% Pro	4.67	5.52	0.007	5.45	0.042
% Leu	10.63	8.91	4 × 10^-6^	9.36	0.046
% Gln	3.92	3.43	0.049	3.19	0.078
% Gly	7.1	7.70	0.099	7.75	0.12
% Glu	5.83	6.46	0.32	6.41	0.22
% Lys	4.31	4.33	0.40	4.54	0.29
% Arg	5.58	5.31	0.58	6.51	0.33
% Asp	5.05	5.89	0.008	5.39	0.38
% Thr	5.35	5.80	0.074	5.58	0.38
% Cys	1.3	0.96	0.01	1.07	0.39
% Tyr	2.79	2.97	0.87	2.83	0.6
% His	2.31	2.12	0.78	2.23	0.62
% Phe	6.12	5.79	0.24	5.75	0.66
% Ala	5.82	5.87	0.92	5.6	0.69
% Asn	3.87	3.98	0.99	3.65	0.93
% Ser	9.37	9.39	0.49	9.29	0.96
% Ile	4.4	4.18	0.54	4.46	0.99
Hydrophobicity	-21	-85	0.049	-93	0.067
SignalP	0.2	*	*	0.2	0.83
NetNGlyc	1.1	1.3	0.52	1.1	0.75
NetOGlyc-S	0.1	0.00	0.57	0	0.29
NetOGlyc-T	0.6	0.7	0.15	0.5	0.97
LCR	0.9	0.9	0.80	0.9	0.84
% α-Helix	42	32	3 × 10^-4^	32	0.015
% β-Sheet	15	21	1.5 × 10^-4^	22	0.002
THMM	1.4	0.3	0.023	0.5	0.009
pI	7.1	7.0	0.67	6.9	0.4

* Not applied as the prediction program as bacterial targets are both gram positive and gram negative.

Targets were found to contain more polar, charged, basic and acidic amino acids. The mean frequency of positively charged amino acids was greater in *E. coli *(14.3%) and in all targets (14%) compared to non-targets (12.5%) (p-value = 0.01 for all targets). The mean frequency of polar amino acids in *E. coli *(45.1%) and all targets (45.3%) was higher compared to non-targets (42.8%) with a significant p-value of 0.0251 for all targets; similarly, there was a higher proportion of non-polar amino acids in non-targets compared to all targets (54.9% in *E. coli*, 57.1% in non-targets, and 54.6% in targets) with a p-value of 0.0281 for all targets. The frequencies of negative amino acids were greater for *E. coli *(11.9%) and all targets (11.9%) compared to non-targets (10.8%) (p-value = 0.070 for all targets).

### Enzyme Class

**Figure 1:**
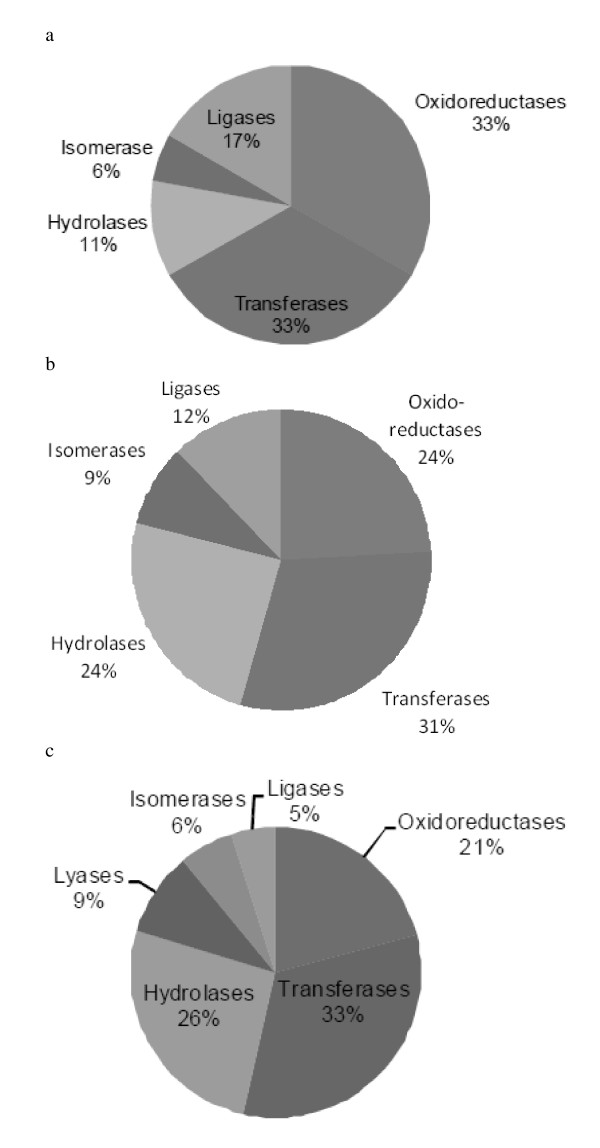
**Distribution of enzyme classes**. a - Distribution of enzyme classes for the *E. coli *targets dataset. b - Distribution of enzyme classes for all bacterial targets dataset. c - Distribution of the enzyme class for the non-targets dataset.

The percentage of enzymes was found to be 33% (1420/4243) in non-targets compared to 81% (18/22) for *E. coli *and 71% (34/48) for bacterial targets. Figure 1 shows high preferences for ligases in bacterial targets (12%) and for *E. coli *(17%), compared to 5% for non-targets. Ligases are common antibacterial drug targets as they are involved in the formation of the cell wall. Lyases were not found in either the bacterial or *E. coli *target sets.

### Subcellular Location

**Figure 2:**
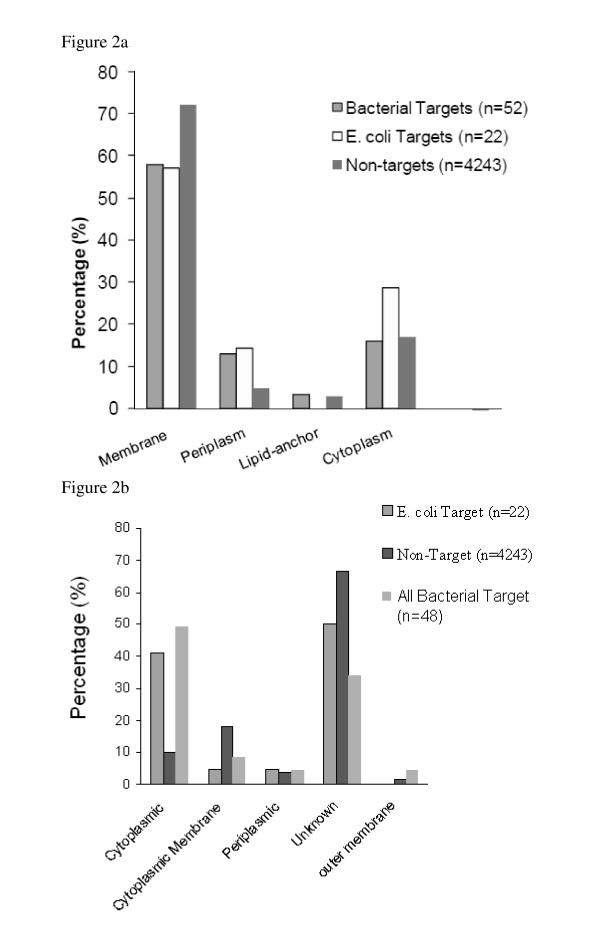
**Subcellular locations**. a - Subcellular locations for targets and non-targets from SwissProt annotations. b - The distribution of subcellular locations for *E. coli *targets and non-targets using the prediction program (PSORTb).

43% (1829/4243) of the non-targets, 40% (9/22) of the *E. coli *targets and 47% (23/48) of the bacterial targets have subcellular location annotations in SwissProt. These percentages might be misleading due to bias of annotated sequences to a specific family or incomplete annotation. Figure 2a shows that the majority of non-drug targets are localised to the bacterial cell membrane. The membrane location includes all entries that contain the word membrane such as *membrane single-pass type II, peripheral membrane protein, peripheral membrane protein, multi-pass membrane, cell membrane and cell inner membrane (potential/by similarity)*. The next highest preferences for drug targets are in the cytoplasm and periplasm.

A prediction method (PSORTb) [10] was used to validate the results of subcellular location. A localization score is calculated for each protein, where the threshold is 7.5 or greater. If two locations have high scores, the prediction is set to unknown. Figure 2b shows the subcellular location results found using PSORTb. The prediction program performs a different analysis depending on whether the organism is gram-positive or gram-negative. Hence the analysis was performed between the two gram-negative datasets of *E. coli *targets and non-targets. Non-targets are localised in the cytoplasmic membrane more than the cytoplasm, and vice versa for *E. coli *targets. Unknown assignments, where the program is unable to make a prediction, are frequent for both *E. coli *targets (50%) and non-targets (65%).

### Gene Ontology

Additional file 1: Supplemental Figures S1a-f show that molecular functions at level 1 and level 2 are similar for bacterial targets, *E. coli *targets and non-targets.

The distribution of biological process at level 1 (Additional file 1: Supplemental Figures S1g-i) shows that terms such as *metabolic process *and *cellular process *are found with similar percentages in bacterial targets, *E. coli *targets and non-targets. *Response to antibiotic *is unsurprisingly found in bacterial targets and *E. coli *targets, but not non-targets. Terms including *localization *and *establishment of localization *are common for non-targets, but they are not associated with bacterial or *E. coli *targets. The distribution of biological process at level 2 (Additional file 1: Supplemental Figures S1j-l) shows that *primary metabolic process *and *cellular metabolic process *have similar frequencies for both targets and non-targets. *The regulation of cell shape *term is present in both *E. coli *(14%) and in all bacterial targets (6%), but no proteins are associated with this process in the non-targets. Other processes favoured for bacterial and *E. coli *targets are *biosynthetic process *and *cell wall organization. Transport proteins *are more prevalent for non-targets.

Within the cellular component class, the *organelle and cell part *terms are present with similar frequencies at level 1 for bacterial targets, *E. coli *targets and non-targets (graph not shown). At level 2, more bacterial and *E. coli *targets are localised in the *intracellular part *compared to non-targets (Additional file 1: Supplemental Figures S1m-o). This class includes *cytoplasm and cytoplasmic part. Intracellular organelle *and *intracellular non-membrane bound organelle *terms are strongly favoured for bacterial and *E. coli *targets compared to non-targets.

### Prediction of New Antibiotic Targets

The features described above were used with the support vector machine algorithm to develop a model that is able to assign any sequence to the *E. coli *target or non-target classes solely from protein sequence. We only used *E. coli *targets versus *E. coli *non-targets, rather than training all targets versus *E. coli *non-targets because differences to all targets may reflect species differences instead of differences between targets and non-targets. Two parameters were varied to maximise the accuracy, namely the error penalty (C) for an incorrect prediction and the radial basis function parameter (γ) which controls how smooth the boundary is in hyperspace between the target and non-target areas. The accuracy is defined as the overall probability that the prediction is correct. A PERL program was coded to search for the optimal C and γ values by performing a coarse grid and a fine grid search. The optimal parameters for C and γ were found to be 0.9 and 0.1, respectively. This model had an accuracy of 94.2% using 32 features including 20 amino acids, length, hydrophobicity, signalP, netNglyc, netOlgyc-S, netOglyc-T, LCR, secondary structure (α and β), TMHMM and pI. This accuracy is based on 5-fold cross validation using 22 *E. coli *targets as the positive training dataset and 200 proteins for the *E. coli *non-targets. The area under curve in a ROC plot was 0.996, confirming the high accuracy of the model.

The optimised Support Vector Machine (SVM) algorithm was used to classify 4243 *E. coli *non-target sequences. 319 of them were assigned as targets, suggesting that 7% of bacterial proteins have target-like properties (Additional File 2). Of these predicted *E. coli *targets, 35% (113/319) are annotated as uncharacterised proteins and 8% (28/319) as 50S or 30S ribosomal proteins. 8% (26/319) of the new predicted targets are transferases and 5% (16/319) are hydrolases, in broad agreement with annotation data (Figure 1a-b). Other EC classes were less abundant, with four isomerases, three oxidoreductases, two lyases and one ligase.

The 319 predicted *E. coli *target proteins were further analysed to check for similarity to human proteins. If any similarity is found, the predicted antibiotic targets may well be problematic, as their inhibition could lead to toxic effects in the patient. 63 proteins were found to have maximal sequence similarities to a human protein of 25% to 63% using BLAST (Additional File 3). Removal of these 63 proteins leaves 256 potential new targets with no significant similarity to any human protein.

**Figure 3:**
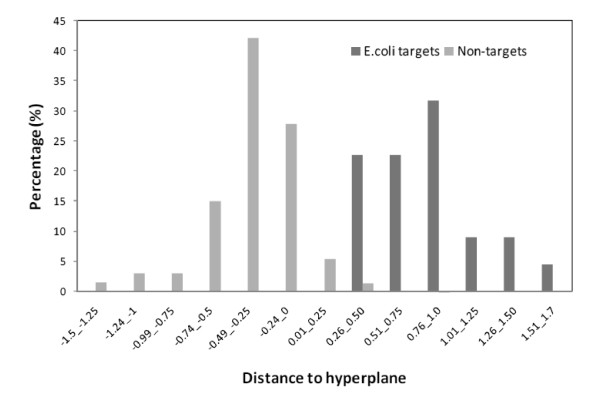
**Prediction confidence distributions of *E. coli*targets and non-targets datasets**.

The distance of a protein from the SVM hyperplane boundary between the two sets (Additional File 2) gives a measure of the accuracy of the distribution of the data about the decision surface. The further the score is from zero, the more reliable is the prediction. Figure 3 illustrates the calculated distance of the *E. coli *targets and non-targets from the hyperplane, in 0.25 intervals. A protein with a positive distance is classified as a target and vice versa. Excellent separation between the two sets is achieved, with all targets classified correctly. The 319 false negatives are the non-targets with a positive score. These are the most interesting proteins, as they are potential new antibiotic targets.

The list of 319 proteins was subjected to a BLAST search against the *E. coli *essential genes. 20% (64/319) of the new predicted targets showed a high similarity to an essential gene where 11 proteins had a sequence identity of 50% to 70% and 53 proteins had a sequence identity > 70%. Additional File 4 shows the matching essential gene for each protein along with their p-values, identities and BLAST scores for each protein. These 64 proteins are more likely to be successful antibiotic targets since their loss of function is likely to lead to cellular growth arrest or death. We do note, however, that there are beneficial *E. coli *colonies in humans, so targeting *E. coli *may have unwanted effects.

Hu *et al. *recently performed a detailed and thorough analysis of orphan *E. coli *proteins, giving many new functional assignments [11]. In particular, they were able to assign 92 orphans to translation and 99 to bacterial cell envelope biogenesis, pathways that are known to be associated with antibiotic modes-of-action. When these are compared to our predicted targets, we find 40 common proteins that are particularly promising targets, specifically: 14 orphan and 9 annotated proteins responsible for cell envelope biogenesis, and 12 orphan and 5 annotated proteins responsible for translation (Additional File 5).

## Discussion

Even though we have only a fairly small number of distinct targets in our data set, we still find many properties that are significantly different between antibiotic targets and non-targets. (The Mann-Whitney U test that we used to determine p-values take the data set sizes into account.) There was little difference between *E. coli *targets and those from all bacteria. Targets are more likely to be enzymes than non-targets. Major antibiotic mechanisms include inhibiting enzymes responsible for translation, transcription, replication and bacterial cell wall biosynthesis. Inhibition of such mechanisms usually results in cell growth inhibition or bacterial death. The molecular function analyses of Gene Ontology and enzyme class data show a preference for ligases as targets, such as those involved in peptidoglycan biosynthesis. The biological process analysis shows that *response to antibiotic, regulation of cell shape *and *cell wall organization *terms are favoured for targets. The cellular components data showed a high preference for *intracellular part *targets, with the *cytoplasm *subdivision favoured in particular, perhaps because enzymes are abundant in the cytoplasm and despite the difficulty of a drug crossing the membrane. The membrane distribution for targets was lower that non-targets, supporting the predicted subcellular locations. While the annotated results had a higher preference for targets to be localised in the membrane, the predicted results had a higher preference for the cytoplasm. There are fewer predicted transmembrane helices in targets, supporting the subcellular location data.

Bacterial targets tend to be larger and more polar than non-targets. Larger proteins have more potential surface of interaction between the drug and the target and may participate in more protein-protein interactions, thus having a larger effect when a drug binds. The presence of charged amino acids might facilitate the interaction between the drug and the target, as bonds to charged amino acids from target groups are common [12-14]. Positively charged proteins are more likely to be ribosomal, since they interact with strongly negatively charged rRNA.

Studies on *E. coli *showed that 35% of metabolic enzymes, including amino acid, purine and pyrimidine biosynthesis, are non-essential genes for cell growth [15]. This means that if one enzyme is knocked out from the pathway, the cell is able to function through alternative routes, including using isoenzymes and multifunction enzymes that can substitute for the missing enzymes. Non-targets favour pathways such as amino acid and macromolecule biosynthesis, while targets show a preference for transcription and DNA replication Gene Ontology (GO) terms. Hence, metabolic enzymes are disfavoured as antibacterial drug targets.

An ideal drug should have no similar protein in the host cell, as the drug might interact with it and lead to adverse effects in the patient. There exist some exceptions for this rule: Trimethoprim is such an example of an antibiotic used to treat urinary tract infections. It works on inhibiting dihydrofolate reductase, despite the presence of a close human homologue [16]. On the other hand, there are no guarantees that if there is no homology, toxicity is not observed. An example of that is the large ribosomal subunit which is targeted by chloremphenicol. In spite of the difference in the structure of the prokaryotic and eukaryotic ribosomes, a functionally conserved area is found in the ribosomes of mitochondria and bacteria resulting in side effects in patients [17].

Our previous work analysed desirable properties in human drug target proteins [18], so we can now compare target proteins in humans and bacteria. The properties differ greatly, reflecting entirely different modes-of-action (e.g. a bacterial drug should ideally kill the host, while this is generally to be avoided in humans, with possible exceptions, e.g. for tumour cells), as well as differences between prokaryotes and eukaryotes. Membrane proteins are common targets in humans, while they are rare as bacterial targets. N-glycosylation is more common in human targets sites, which implies that human targets proteins have a relative longer lifetime than bacterial targets and human non-targets. Human targets are frequently enzymes involved with binding and signaling; bacterial targets are often enzymes, involved in protein binding or ribosomal proteins. The most targeted bacterial biological processes are peptidoglycan biosynthesis and cell wall synthesis, compared to cell communication and regulation of biological process for human targets.

In summary, the following seem to be desirable features for an antibiotic drug target:

• Essential for the survival of the bacterial cell

• No close human homologue

• Must be present in a number of pathogens if broad-spectrum action is required. Narrow spectrum antibiotics target specific pathogens

• The most targeted biological processes are peptidoglycan biosynthesis, cell wall synthesis, transcription and translation

• Capable of binding to a small molecule, implying the presence of a binding site

• Favoured to be localized in the cytoplasm and less frequent in membranes

• Favoured to be ribosomal proteins

• More likely to be enzymes, transferases, hydrolases or ligase, but not lyases. Although enzymes are highly targeted, metabolic enzymes are disfavoured for antibacterial drug targets.

While many of the above properties have been reported in qualitative terms, they have not been previously quantified, to our knowledge. In addition, some of the preferences we find appear to be new, such as EC class, length, amino acid frequencies, post-translational modifications, secondary structure contents, pI, SignalP and transmembrane helices.

The machine learning work can accurately assign an *E. coli *protein to the target or non-target classes. The use of cross-validation and the generalisation parameter in the SVM ensures to a certain extent that the model can generalise to data not used in the training process. After classifying the entire *E. coli *proteome to identify novel target-like proteins and pruning it to remove those that are similar to a human protein, we find 256 proteins that may be potential antibiotic targets, especially those 64 proteins highly similar to essential genes. While 40 previously identified orphan genes can now be assigned to cell envelope biogenesis or translation, many are currently of unknown function and thus deserve further investigation. The rules listed above and our list of potential new antibiotic targets may help in the identification of new antibiotics drug target.

## Conclusions

Comparison of antibiotic protein targets with non-target proteins from *E. coli *has allowed the identification of a number of properties that are desirable in drug targets. Even though the number of unique known targets is not large, statistically significant differences in property frequencies were found. Using sequence features for machine learning allows accurate identification of targets, as shown by a cross-validation accuracy of 94%. Applying the optimised support vector machine to the *E. coli *proteome identifies hundreds of proteins that have similar properties to known antibiotic drug targets. These proteins may therefore be considered as potential new targets for novel antibiotics.

## Methods

### Data sets

The bacterial target dataset was downloaded from DrugBank [19] in February 2008. The bacterial dataset consists of 66 bacterial targets from different species and strains. The set was culled with the PISCES program [20] so that no two proteins had more than 20% sequence identity to give a list of 48 non-redundant bacterial targets. By far the most common species within which antibiotic targets are found is *E. coli *(strain K-12). This *E. coli *targets dataset consists of 22 proteins. The non-targets dataset was downloaded from the High-quality Automated and Manual Annotation of microbial Proteomes (HAMAP) at Expasy http://www.expasy.ch/sprot/hamap/. It is not obvious what a bacterial non-target protein data set should be, as it is always arbitrary which species are included. We therefore picked just one species, namely *E. coli *strain K-12, as this is the most common species for targets. As of 2006, the *E. coli *proteome contains highly accurate and complete sequences for the K-12 strain with 4338 entries. This set was culled with a 20% sequence identity cut-off to leave 4265 entries for the non-redundant dataset. Known antibiotic drug targets were removed from the set to give a non-redundant non-targets dataset of 4243 entries. Full lists of bacterial targets and non-targets data sets are found in Additional File 6.

When comparing all targets to non-targets, differences between the sets may arise since the targets are taken from a wide range of bacteria and they are compared only to one *E. coli *strain. Hence, we may be picking up differences in protein features arising from differences between species, rather than between targets and non-targets. Comparing targets with non-targets entirely within the *E. coli *K-12 strain avoids this problem, though the number of targets is considerably fewer. We therefore analysed three data sets, namely all antibiotic targets, *E. coli *K-12 strain targets and *E. coli *K-12 strain non-targets. The non-drug target dataset may contain proteins that could be drug targets, making it less well defined. However, as it is doubtful whether more than a small fraction of the bacterial proteome will ever be a target, this error should not be large.

Properties were tested for normality using the Kolmogorov-Smirnov test. As all the distributions were not normal, the Mann-Whitney U test was used to test for statistically significant differences between the sets using SPSS (Statistical Package for Social Sciences).

SWISS-PROT annotations were used for some sequence properties, such as sub-cellular locations. We were concerned, however, that variations in the frequency of annotation may bias the results. For example, one would expect that drug target proteins are better studied than non-targets, leading to a possible increase in annotation of targets compared to non-targets. We therefore also often used predicted properties, as well as annotations, where possible.

### Simple sequence properties

The frequency of each amino acid in each protein is divided by protein length to give percentage frequencies. Amino acids were grouped as follows: non-polar (A, C, F, G, I, L, M, P, V, W, Y); polar (D, E, H, K, N, Q, R, S, T, Z); positive (H, K, R) and negative (D, E). Hydrophobicity was calculated as the sum of hydrophobicity values using the Kyte & Doolittle index [21], divided by the number of residues in each of the protein sequences. The Pepstats program http://emboss.cbr.nrc.ca/cgi-bin/emboss/pepstats was used to output protein sequence information statistics including isoelectric points (pI values), and numbers of positively charged and negatively charged amino acids. A PERL script was written to extract protein sequence features and calculate the mean frequencies.

### EC number

Primary EC numbers were taken from the description line "DE", as this line contains the EC numbers corresponding to an entry, where primary EC numbers 1-6 are oxidoreductase, transferase, hydrolase, lyase, isomerase and ligase, respectively.

### Gene Ontology terms

Gene Ontology (GO) provides a controlled vocabulary to describe gene function [22], using the following three organising principles: (a) biological process; (b) molecular function; (c) cellular component. The GO database was downloaded from http://www.geneontology.org/GO.downloads.shtml, dated September 2006. GO ID annotations are in SWISS-PROT in the database reference line "DR". A PERL program was coded to extract the Gene Ontology IDs for each datasets and to parse a GO file and write child/parent relationship as a full path for every GO term and to output the highest level and next highest level for every path as levels 1 and 2. If a GO term id was present more than once at level 1 or 2, it was only counted once at that level.

### Predicted Sequence Features

The NetOglyc program http://www.cbs.dtu.dk/services/NetOGlyc/ was used for identification of *O*-glycosylation sites using neural networks [23]. The NetNglyc program http://www.cbs.dtu.dk/services/NetNGlyc/ was used for identification of *N*-glycosylation sites [24]. The WoLF PSORTB program http://wolfpsort.seq.cbrc.jp/[25] was used to predict protein subcellular localization. It makes predictions based on known sorting signal motifs and sequence features, such as amino acid content. The output report of WoLF PSORTB was analysed by extracting the location of the highest predicted location. The SignalP program http://www.cbs.dtu.dk/services/SignalP/ was used to perform signal peptide prediction [26]. The TMHMM method http://www.cbs.dtu.dk/services/TMHMM/[27] was used to predict the location and orientation of α-helices in membrane-spanning proteins. The SEG program [28] was downloaded from ftp://ftp.ncbi.nih.gov/pub/seg/seg/ to detect low complexity regions. It is used to mask composition-biased regions in the query, based on a statistical approach. The JPred program http://www.compbio.dundee.ac.uk/~www-jpred/[29] was used to predict the percentages of α-helix and β-sheet. Accurate secondary structure could have been found from crystal structures. However, most proteins do not have structures, particularly if they are membrane proteins. We therefore used a secondary structure prediction program, rather than using structures.

### Machine Learning

Support vector machines [30], using a radial basis function, were used to make a classifier that can distinguish *E. coli *targets from non-targets, using features that can be calculated from any protein sequence. The features we used were: amino acid compositions, length, hydrophobicity, SignalP, NetOglyc Ser, NetOglyc Thr, low complexity regions, α-helix, β-sheet, transmembrane helices and pI. A scaling scheme was used for every vector by restricting all entries to be between 0 and 1, by calculating for every feature (X - Min)/(Max - Min) where X is the feature score, and Min and Max are the minimum and maximum values of X in the set.

The SVM was assessed using 5-fold cross validation using the following training set splits: 22 *E. coli *targets were used as the positive training dataset and 200 proteins were randomly selected from the *E. coli *non-targets dataset to represent the negative set. This model is therefore based on training *E. coli *targets versus non-targets, rather than training all targets versus non-targets because differences to all targets may reflect species differences instead of differences between targets and non-targets. As the target and non-target sets have different sizes, an error penalty parameter (C) is introduced to ensure generalization of the classifier. The ratio of the error penalty for targets:non-targets is set to 9:1, in proportion to the data set sizes of 200:22 and applied in the LIBSVM program using the weight parameter (wi) for both classes (1,-1). The program (svm-predict-margins) was downloaded from http://www.work.caltech.edu/~htlin/program/libsvm/#margin[31]. This tool calculates the unnormalised distance from the SVM hyperplane.

### Essentiality

Essential genes are those that play an important and essential functional role in a specific pathway. The presence of these genes is vital to the cell, as their inhibition would lead to cellular growth arrest or death of the pathogen. Essential genes are those that are not able to recover from a random insertion disruption [32]. *E. coli *essential genes were downloaded from the National Microbial Pathogen Data resource (NMPDR) http://www.nmpdr.org/FIG/wiki/view.cgi/Main/EssentialGenes. The database consists of 603 non-redundant putative essential genes, updated in September 2008. The list is checked for any redundancy using BLASTCLUST[33], so that no pairs of sequences had a sequence identity of more than a strict cut-off of 20%. The resulting list can be found in Additional File 7.

## Authors' contributions

TB participated in the design of the study, carried out the work and helped to draft the manuscript. AD conceived of the study, participated in its design and coordination and helped to draft the manuscript. Both authors read and approved the final manuscript.

## Supplementary Material

Additional file 1 **Gene Ontology Terms**. Pie charts for frequencies of Gene Ontology terms for *E. coli *targets, bacterial targets and non-targets. Supplemental Figure 1a - Distribution of molecular functions at level 1 for *E. coli *targets. Supplemental Figure 1b - Distribution of molecular functions at level 1 for bacterial targets. Supplemental Figure 1c - Distribution of molecular functions at level 1 for non-targets. Supplemental Figure 1d - Distribution of molecular functions at level 2 for *E. coli *targets. Supplemental Figure 1e - Distribution of molecular functions at level 2 for bacterial targets. Supplemental Figure 1f - Distribution of molecular functions at level 2 for non-targets. Supplemental Figure 1g - Distribution of biological processes at level 1 for *E. coli *targets. Supplemental Figure 1h - Distribution of biological processes at level 1 for bacterial targets. Supplemental Figure 1i - Distribution of biological processes at level 1 for non-targets. Supplemental Figure 1j - Distribution of biological processes at level 2 for *E. coli *targets. Supplemental Figure 1k - Distribution of biological processes at level 2 for bacterial targets. Supplemental Figure 1l - Distribution of biological processes at level 2 for non-targets. Supplemental Figure 1m - Distribution of cellular components at level 2 for *E. coli *targets. Supplemental Figure 1n - Distribution of cellular components at level 2 for bacterial targets. Supplemental Figure 1o - Distribution of cellular components at level 2 for non-targets. Click here for file

Additional file 2 **New predicted antibiotic targets**. *E. coli *proteins with target-like properties. Results from applying SVM trained on distinguishing bacterial targets from non-targets. Click here for file

Additional file 3 **Predicted bacterial protein targets with sequence similarity to human proteins**. Predicted bacterial protein targets (Additional File 2) were run with BLAST against the human proteome. All significant sequence matches are recorded here. Click here for file

Additional file 4 **New predicted targets with high similarity to essential genes**. Predicted bacterial protein targets was subjected to a BLAST search against the *E. coli *essential genes. The 64 targets that have a high similarity to an essential gene are listed here, along with their p-values, identities and BLAST scores. Click here for file

Additional file 5 **Comparison to global functional atlas of *E. coli***. Hu *et al. *analysed *E. coli *orphan proteins, giving many new functional assignments [11]. In particular, they assigned 191 to categories associated with antibiotic drug targets. Here we compare our predicted target proteins to this global functional atlas, giving 40 common proteins that are particularly promising targets. Click here for file

Additional file 6 **Bacterial targets and non-targets data sets**. Non-redundant bacterial targets and non-targets data sets. Click here for file

Additional file 7 **Non-redundant putative essential genes**. Essential genes are those that are not able to recover from a random insertion disruption [32]. 603 non-redundant putative essential genes are listed here, downloaded from the National Microbial Pathogen Data resource (NMPDR) http://www.nmpdr.org/FIG/wiki/view.cgi/Main/EssentialGenes. Click here for file
