# Supplementary material for: Properties and identification of antibiotic drug targets
Source: BMC Bioinformatics. 2010 Apr 20;11:195. doi: 10.1186/1471-2105-11-195 (PMC2873537; doi:10.1186/1471-2105-11-195)
Supplement: Additional file 1 — Gene Ontology Terms. Pie charts for frequencies of Gene Ontology terms for E. coli targets, bacterial targets and non-targets. Supplemental Figure 1a - Distribution of molecular functions at level 1 for E. coli targets. Supplemental Figure 1b - Distribution of molecular functions at level 1 for bacterial targets. Supplemental Figure 1c - Distribution of molecular functions at level 1 for non-targets. Supplemental Figure 1d - Distribution of molecular functions at level 2 for E. coli targets. Supplemental Figure 1e - Distribution of molecular functions at level 2 for bacterial targets. Supplemental Figure 1f - Distribution of molecular functions at level 2 for non-targets. Supplemental Figure 1g - Distribution of biological processes at level 1 for E. coli targets. Supplemental Figure 1h - Distribution of biological processes at level 1 for bacterial targets. Supplemental Figure 1i - Distribution of biological processes at level 1 for non-targets. Supplemental Figure 1j - Distribution of biological processes at level 2 for E. coli targets. Supplemental Figure 1k - Distribution of biological processes at level 2 for bacterial targets. Supplemental Figure 1l - Distribution of biological processes at level 2 for non-targets. Supplemental Figure 1m - Distribution of cellular components at level 2 for E. coli targets. Supplemental Figure 1n - Distribution of cellular components at level 2 for bacterial targets. Supplemental Figure 1o - Distribution of cellular components at level 2 for non-targets. [file 1471-2105-11-195-S1.DOC]

**Gene Ontology Terms**

Figure 1a The distribution of molecular functions at level 1 for *E. coli* targets

Figure 1b The distribution of molecular functions at level 1 for bacterial targets.

Figure 1c The distribution of molecular functions at level 1 for non-targets.

Figure 1d The distribution of molecular functions at level 2 for *E. coli* targets

Figure 1e The distribution of molecular functions at level 2 for bacterial targets.

Figure 1f The distribution of molecular functions at level 2 for non-targets.

*Figure 5.8 The distribution of molecular functions at level 2 targeted by bacterial targets (top) and the non-targets dataset (bottom).*

Figure 1g The distribution of biological processes at level 1 for *E. coli* targets

Figure 1h The distribution of biological processes at level 1 for bacterial targets.

Figure 1i The distribution of biological processes at level 1 for non-targets.

Figure 1j The distribution of biological processes at level 2 for *E. coli* targets.

Figure 1k The distribution of biological processes at level 2 for bacterial targets.

Figure 1l The distribution of biological processes at level 2 for non-targets.

Figure 1m The distribution of cellular components at level 2 for *E. coli* targets.

Figure 1n The distribution of cellular components at level 2 for bacterial targets.

Figure 1o The distribution of cellular components at level 2 for non-targets.
